# Supplementary figures and images for: Vaporous Marketing: Uncovering Pervasive Electronic Cigarette Advertisements on Twitter
Source: PLoS One. 2016 Jul 13;11(7):e0157304. doi: 10.1371/journal.pone.0157304 (PMC4943591; doi:10.1371/journal.pone.0157304)

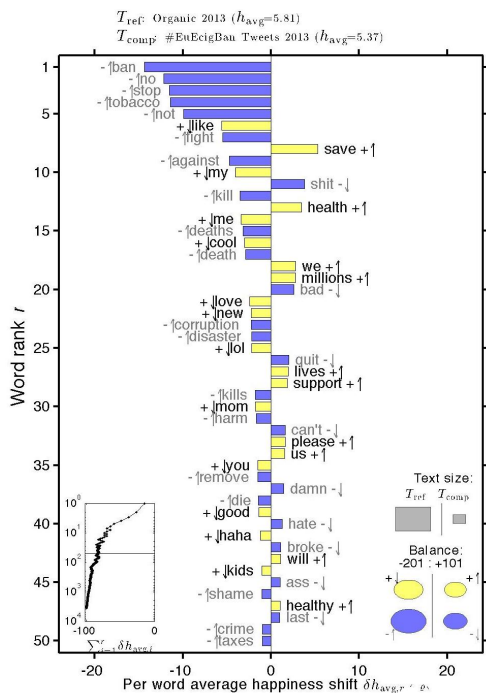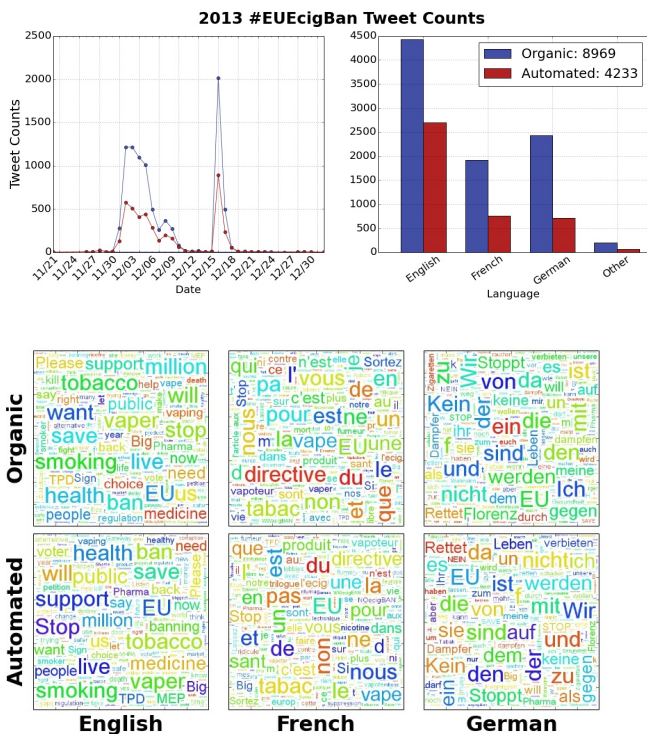

Supplement: S1 Fig — (Left) Word shift graph comparing tweets tagged #EUecigBan against 2013 English Organic User Tweets (untagged). (top-right) The automated and Organic tagged tweet distributions are plotted. A histogram displays the counts per language and user class. (bottom-right) Word clouds compare ranked-word frequencies across language and user type. Each categorical time-series exhibits a severe negative trend occurring between December 2013 and January 2014. There is an inverse relationship with the average happiness scores during this time period. This was during the time that the EU was debating strict regulation and a possible ban on specific e-cigarette products [12]. Hashtags (#) allow users to categorize the content of their tweets. During this period, 13,227 sampled tweets were tagged with #EUecigBan. In S1 Fig, a word shift graph (left) visualizes the sentiments from English Organic users using #EUecigBan versus the remaining Organic tweets from 2013. English Tweets tagged #EuEcigBan are the comparison distribution in reference to all other tweets from 2013. Tweets containing #EuEcigBan are on average much more negative (havg 5.81 versus 5.37) due to an increase in the negative words ‘ban’, ‘stop’, ‘no’, ‘not’, ‘fight’, ‘against’, ‘disaster’, ‘death’, ‘corruption’, ‘tobacco’, ‘kills’, etc. The positive words also disfavor the legislation, with the words ‘save’, ‘millions’, ‘lives’, ‘support’, ‘healthy’ occurring more frequently. English, French, and German tagged tweets were the most prevalent, and word clouds help visualize themes between language and user class. This shows that Twitter sentiments can be useful in gauging public opinion toward regulation of electronic cigarettes. There is also a heavy automated tweet presence in each language with a similar attitude regarding the legislation, as depicted in the word clouds. Future work should also investigate if and how automated users can impact organic opinion on legislation. (PDF) [file pone.0157304.s001.pdf]
