# Supplementary material for: Vaporous Marketing: Uncovering Pervasive Electronic Cigarette Advertisements on Twitter
Source: PLoS One. 2016 Jul 13;11(7):e0157304. doi: 10.1371/journal.pone.0157304 (PMC4943591; doi:10.1371/journal.pone.0157304)
Supplement: S1 Table — List of all key words used in the analysis. Flavors compiled from https://crazyvapors.com/e-liquid-flavor-list/ Keywords other than ‘General Twitter Scrape’ were applied to categorize automated account tweets. (PDF) [file pone.0157304.s002.pdf]

S1 Table: Key Words for Data Collection and Categorical Analysis

Type	Keywords
**General Twitter Scrape** (includes hashtag variants)	ecig, e cig, e-cig, ecigs, e cigs, e-cigs, e ciggs, e ciggs, e-ciggs, eciggs, e cigg, ecigg, e-cigarette e cigarette, e cigarettes, e-cigarettes, electronic cigarette blucigs, blucig, blu cig, blu cigs, blu ciggs, electronic cigarettes
**Commercial**	buy, save, coupon, coupons, discount, price, cost, deal, promo, money, sale ,purchase, offer, review, code ,win, winner, starter kit, starter kits, premium, $, kit, %, sales,voucher, brand, free e cigarette, free electronic cigarette, free e cig, free ecig
**Cessation**	quit, quitting, quits, stop smoking, smoke free, quitter, safe, safest, safer, quitsmoking, give up smoking
**Discount**	free trial, free shipping, free sample ,free samples, coupon, discount, discounts, save, sale, coupons, deal, deals, free e cigarette, free electronic cigarette, free e cig, free ecig
**Flavors***	flavor, flavour, flavors, flavours, flavored, flavoured Cherry, Lime, Almond Coconut Bar, Alpine Fresh, Amaretto, Apple Pie (Ala Mode), Banana, Banana Cream, Banana Graham, Banana Nut Bread ,Banana Pudding, Banana Split, Bavarian Cream, Belgian Waffle Berry Blast, Black Cherry, Black Berry, Black Honey, Blazing Frost, Blueberry,Blueberry Cheesecake, Blueberry Cinnamon Crumble, Blueberry Cotton Candy Blueberry Delight,Brandy, Bubble Gum, Butterscotch Butter Rum, Buttered Popcorn, Cafe Latte, Cake Batter, Candy Cane, Candy Apple, Cantaloupe, Caramel Caramel Cappuccino, Cappuccino,Champagne, Cheesecake, Chocolate Covered Raspberries

Electronic Cigarette Table of Key Words Continued

Type	Keywords
**Flavors* (continued)**	Cinnamon Coffee Cake, Cinnamon Danish,
	Cinnamon Sugar Cookie, Circus Cotton Candy
	Clove, Coconut, Coconut Candy, Coffee
	Coffee&Cream, Cola, Cool, Cotton Candy
	Cranberry, Crazy Berry, Crazy Chill, Crazy Dew
	Crazy Freeze, Crazy Grass, Crazy Hump
	Crazy Pep, Crazy Rainbow, Crazy Watermelon
	Cream Cheese Frosting, Cream de Menthe
	Creamy Fruit Smoothie, Cuban Cigar
	Cured TobaccoDaquiri, DK-Tab, Double Chocolate
	Dragon's Blood, Dragon Fruit, Dulce De Leche
	Egg Nog, English Toffee, Espresso, Extreme Ice
	Flaming Peach, French Toast, French Vanilla,
	French Vanilla Deluxe, Fresh Apple, Fresh-N-Fruity
	Fudge Brownie, Fruit Rocket, Georgia Peach, Gingerbread
	Goblin Goo, Golden Pineapple, Graham Cracker, Green Apple
	Green Tea, Gummy Candy, Harvest Berry, Hazelnut,
	Hot Chocolate, Hot Cinnamon Candy, Hypnotic, Irish Cream,
	Island Getaway, Jamaican Rum, Java Shake, Jungle Juice,
	Kentucky Bourbon, Kettle Corn, Khaluah & Cream, Kiwi,
	Lemon Drop, Lemon Lime, Lemon Meringue Pie, Mango,
	Marshmallow, Melon, Menthol, Mint Patty, Milk Chocolate,
	M-Mix Menthol, M-Mix Special Blend, Mocha, Mojito, Mummy [illegible]
	Munster, N-Mix, N-Mix Menthol, NY Cheesecake,
	Orange Creamsicle, P-Mix, P-Mix Menthol, Papaya
	Passion Fruit, Peanut Butter, Peanut Buttercup,
	Honey Dew Melon, Margarita, M-Mix, Orange Cognac

S1 Table : Electronic Cigarette Table of Key Words
List of all key words used in the analysis. Flavors compiled from `https://crazyvapors.com/e-liquid-flavor-list/` Keywords other than 'General Twitter Scrape' were applied to categorize automated account tweets.
